# Elucidation of a Novel Pathway through Which HDAC1 Controls Cardiomyocyte Differentiation through Expression of SOX-17 and BMP2

**DOI:** 10.1371/journal.pone.0045046

**Published:** 2012-09-12

**Authors:** Eneda Hoxha, Erin Lambers, John A. Wasserstrom, Alexander Mackie, Veronica Ramirez, Tatiana Abramova, Suresh K. Verma, Prasanna Krishnamurthy, Raj Kishore

**Affiliations:** Feinberg Cardiovascular Research Institute, Feinberg School of Medicine, Northwestern University, Chicago, Illinois, United States of America; University of Cincinnati, United States of America

## Abstract

Embryonic Stem Cells not only hold a lot of potential for use in regenerative medicine, but also provide an elegant and efficient way to study specific developmental processes and pathways in mammals when whole animal gene knock out experiments fail. We have investigated a pathway through which HDAC1 affects cardiovascular and more specifically cardiomyocyte differentiation in ES cells by controlling expression of SOX17 and BMP2 during early differentiation. This data explains current discrepancies in the role of HDAC1 in cardiovascular differentiation and sheds light into a new pathway through which ES cells determine cardiovascular cell fate.

## Introduction

Since they were first isolated over a decade ago, ES cells have paved the way for exciting new discoveries [1]. It is through studying the molecular circuitry of ES cells that we have been able to learn key factors that govern pluripotency and differentiation [2], [3] [4]–[6].

HDAC1 has been widely studied due to its implication in many disorders and has been shown to be important during development [7], [8]. HDAC1 knockout mice are embryonic lethal, however cardiac restricted knockout of HDAC1 under the alpha-MHC promoter does not show any deficiencies in heart structure and function at baseline [8]. This has led to the belief that HDAC1 and HDAC2 have redundant roles during differentiation in the heart [8]. Other research investigating the role of HDACs, also points at a possible redundancy between different HDACs. However, most of the current work on HDACs has been done using chemical inhibitors of these enzymes that are not specific to any one HDAC in particular and weekly class specific [9], [10]. A possible redundancy in the role of HDAC1 and HDAC2, however, cannot explain the severe phenotype observed in the global knockout. Additionally, it is not clear at what stage during development HDAC1 is important, so tissue restricted KO of this gene might bypass the stage in which HDAC1 is important and fail to recognize and understand its role. In fact, alpha-MHC is expressed at a very late point in cardiomyocyte development and is more of a maturation marker than a marker for commitment towards the cardiomyocyte phenotype. ES cells are very efficient and useful models to study developmental pathways that cannot be clearly elucidated through the use of KO mice. Because of the apparent discrepancy described in earlier published data for the role of HDAC1, we investigated a possible role for this enzyme in mES cell early differentiation into the cardiovascular cell lineage and elucidated a pathway through which HDAC1 controls cardiomyocyte differentiation. Data presented in this manuscript sheds new light into the cardiomyocyte differentiation circuity of ES cells.

## Results and Discussion

**Figure 1:**
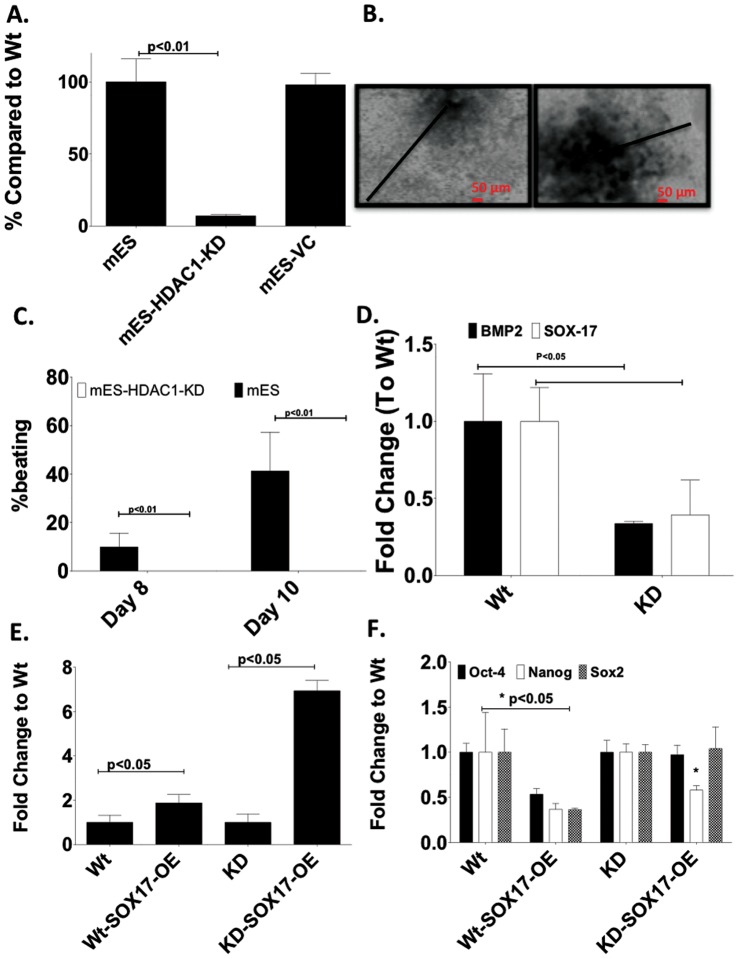
HDAC-1-knockdown mouse ES cells show reduced differentiation and beating ability. A. shRNA was used to create a stable, selectable HDAC1-KD ES cell line. **B.** Light microscopy images showing lack of differentiation in EBs derived from mES-HDAC1-KD cells compared to wt ES cells at day 6 of differentiation. Black arrows indicates distance from the center of the EB to the periphery. **C.** HDAC1-KD-ES cells fail to show any spontaneous beating. **D.** Expression of Sox17 and BMP2 is significantly lower in cells in which HDAC1 has been knocked down compared to wt cells. **E.** Expression levels of Sox-17 mRNA in wt mES and mES-HDAC1-KD cells. **F.** Expression levels of pluripotency-associated genes mRNA in mES and mES-HDAC1-KD cells and mES and mES-HDAC1-KD cells in which SOX-17 has been ectopically expressed.

To elucidate the role of HDAC1 in mES cells in early differentiation and to investigate any cell type specific effects of HDAC1, we created shRNA-mediated stable HDAC1-knock down (HDAC1-KD) cell lines in ES cells (Fig. 1A).

Based on the discrepancy for the role of HDAC1 in the development of the heart observed in previous published work, we hypothesized that HDAC1 played a key role very early in differentiation, before cardiac markers were expressed and was needed for these early specification genes to be expressed. Thus, we investigated the role of HDAC1 in the differentiation of pluripotent cells *in vitro*. We were particularly interested in determining the stage during cardiovascular differentiation at which HDAC1 was important and the pathway through which it induced cardiovascular differentiation.

We investigated the molecular pathway through which HDAC1 was affecting expression of downstream transcription factors important for cardiovascular differentiation. We induced differentiation through Embryoid Body (EB) formation in both wild type (wt) ES cells and in ES cells in which HDAC1 had been stably knocked-down (ES-HDAC1-KD). ES-HDAC1 KD cells failed to expand and did not show any spontaneous beating after differentiation had been induced (Fig. 1A–B). In fact while 40% of EBs derived from wt ES cells show spontaneous beating, none of the ES-HDAC1 KD derived EBs do, even when followed for 26 days into differentiation (Fig. 1B).

Because of the disparate phenotypes of mice with systemic HDAC1 KO and alpha-MHC-driven cardiac restricted HDAC1 deletion, we hypothesized that HDAC1 is important in the regulation of a cardiogenic protein that is expressed very early on, before alpha-MHC, and the expression of which is directly regulated by pluripotency-associated genes. We investigated expression of molecules important in early differentiation that were regulated by pluripotency-associated genes while at the same time could auto-regulate pluripotency gene expression themself. One such gene is Sox-17, the expression of which is significantly reduced in HDAC1-KD cells during differentiation (Fig. 1D). As a differentiation-associated molecule, expression of Sox-17 is suppressed in pluripotent cells by pluripotency-associated genes [11]–. Previous reports have shown an important role of Sox-17 in cardiovascular differentiation and in the regulation of BMP2 expression [14]. We hypothesized that HDAC1 was necessary in ES cells differentiation to cardiovascular lineages in order to repress pluripotency-associated genes, such as Oct4 and Sox2 and thereby allowing for Sox-17 to be expressed. As Sox-17 started to be expressed it independently induced repression of pluripotency-associated genes and expression of BMP2 allowing for the cells to differentiate normally. To elucidate if Sox17-BMP2 regulation was defective in ES HDAC1-KD cells and whether this defect could be corrected, we designed two experimental approaches. We overexpressed Sox-17 in wt and HDAC1-KD ES cells (Fig. 1E), and induced differentiation in these cells through the formation of EBs. Expression levels of pluripotency associated genes did not change in ES cells overexpressing Sox-17 indicating that this molecule alone, is not able to induce differentiation (Fig. 1F). Alternatively, we treated wt and HDAC1-KD derived EBs with an experimentally determined optimal dose of BMP2 during the first two days of differentiation and evaluated morphology, spontaneous contraction and the expression of cardiomyocyte specific transcripts in all cell types. We observed whether exogenous expression of Sox-17 or supplementation with BMP2 would be sufficient to bypass the need for HDAC1, once differentiation had been induced.

**Figure 2:**
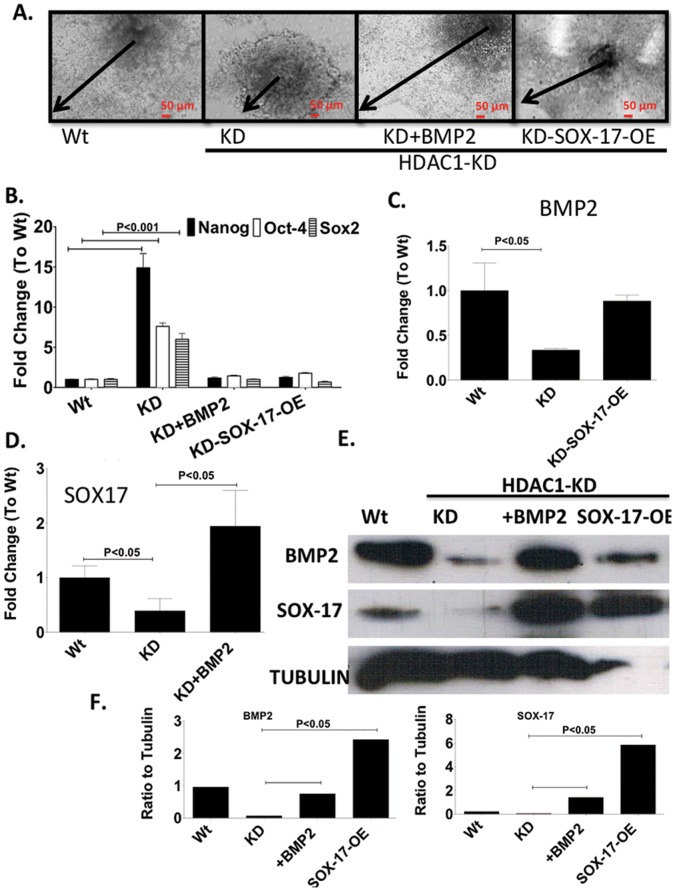
Sox-17 or BMP2 fully rescue HDAC1 phenotype. A. Light microscopy images showing lack of differentiation in EBs derived from mES, mES-HDAC1-KD and mES-HDAC1-KD overexpressing Sox-17 or treated with BMP2 for two days, at day 6 of differentiation. Black arrows indicates distance from the center of the EB to the periphery. **B.** RT-PCR showing expression levels of Sox-2, Oct4 and Nanog on day 6 of differentiation in Wt mES cells mES-HDAC1-KD cells and mES cells lacking HDAC1 over expressing Sox-17 or treated with BMP2 for two days. **C.** RT-PCR showing expression levels of BMP2 in wt mES cells mES-HDAC1-KD cells and mES cells lacking HDAC1 over espressing Sox-17. **D.** RT-PCR showing expression levels of Sox-17 in Wt mES cells mES-HDAC1-KD cells and mES cells lacking HDAC1 over treated with BMP2 for two days. **E.** Western Blot of SOX-17 and BMP2 in Wt mES cells mES-HDAC1-KD cells and mES cells lacking HDAC1 over expressing Sox-17 or treated with BMP2 for two days. Band intensity was quantified using ImageJ. Data in all panels are represented as mean +/− SD and n = 3.

As observed before, mES HDAC1-KD cells showed little expansion of the periphery of EBs. Interestingly however, when Sox-17 was over-expressed in these cells, or when these cells were treated with BMP2, they showed very similar phenotypic and differentiation characteristics as was the case with wt mES cells (Fig. 2A). Additionally, in HDAC1-KD cells in which Sox-17 had been over expressed or had been treated with BMP2 during differentiation, expression levels of pluripotency-associated genes were similar to wt ES cell levels (Fig. 2B), indicating that expression of Sox-17 either through overexpression or through induction from BMP2 was able to repress expression of pluripotency genes independent of HDAC1 during EB differentiation. Both Sox-17 and BMP2 were able to induce expression of each other in HDAC1-KD ES cells to levels similar of these molecules in wt cells (Fig. 2C–E).

**Figure 3:**
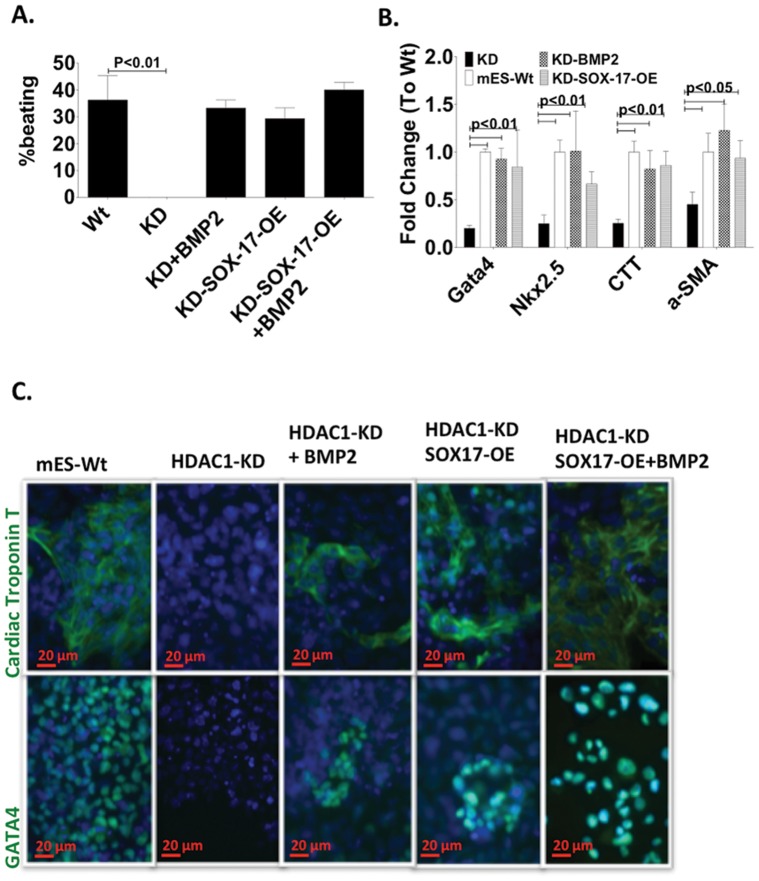
SOX-17 and BMP2 restore expression of cardiomyocyte specific markers in mES HDAC1-KD cells. A. Percentage of beating EBs/total EBs in BMP2 in Wt mES cells mES-HDAC1-KD cells and mES cells lacking HDAC1 over expressing Sox-17 or treated with BMP2 for two days. (n = 3, total EB counted ∼500). **B.** RT-PCR showing expression levels of cardiovascular specific mRNA at day 12 of differentiation in in BMP2 in wt mES cells and mES-HDAC1-KD cells and mES cells lacking HDAC1 over expressing Sox-17 or treated with BMP2 for two days. **C.** Immunofluorescence showing expression levels of GATA4, Cardiac Troponin I (CTI) at day 12 of differentiation in in BMP2 in Wt mES cells mES-HDAC1-KD cells and mES cells lacking HDAC1 over expressing Sox-17 or treated with BMP2 for two days.

Next, we investigated whether supplementing the cells with BMP2 during early differentiation or the overexpression of Sox-17 were enough to fully rescue the HDAC1 phenotype in ES HDAC1-KD cells. As expected, the number of EBs showing beating loci after the HDAC1-KD cells had been treated with BMP2 or in which Sox-17 had been over-expressed were almost identical to wt cells (Fig. 3A, Videos S1, S2, S3, S4). RNA Expression of cardiomyocyte genes was significantly reduced in HDAC1-KD derived differentiating cells and was absent at the protein level (Fig. 3B–C). However, Expression levels of cardiomyocyte genes were restored to wt levels at both RNA and protein level in cells over-expressing Sox-17 or that had been treated with BMP2 (Fig. 3B–C).

**Figure 4:**
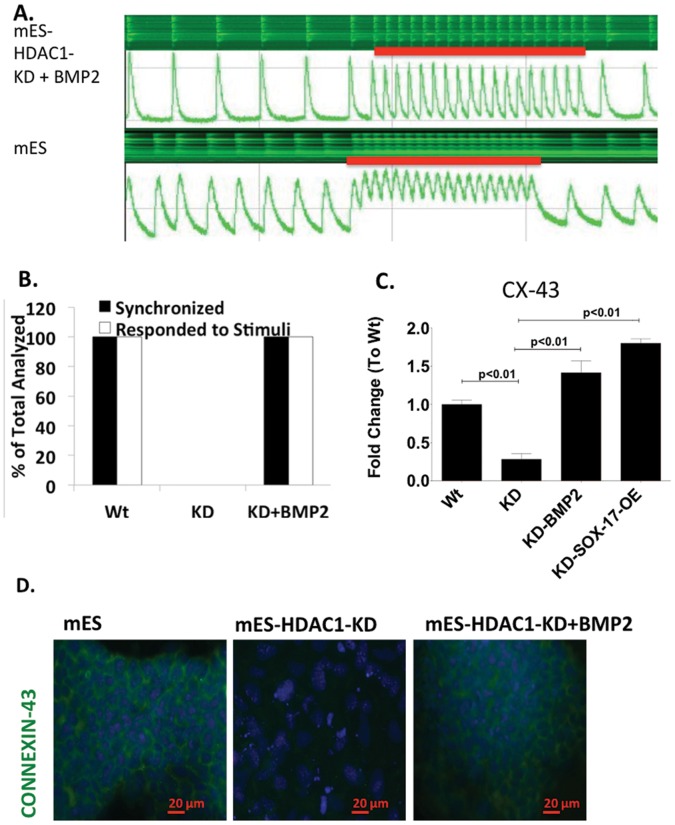
SOX-17 over-expression or BMP2-supplementation is sufficient to to resyore calcium transients and synchrony in HDAC1-KD ES cells. **A.** Calcium uptake and release experiments indicating synchrony and response to stimuli of beating EBs in mES, mES-HDAC1-KD, mES-HDAC1-KD embryoid bodies treated with BMP2 for the first two days of differentiation. **B.** Percentage of EBs that respond to external stimuli and are synchronized (example shown in A). **C.** RT-PCR showing mRNA expression levels of Connexin-43 and **D.** organization of Connexin 43, in mES, mES-HDAC1-KD derived embryoid bodies and mES-HDAC1-KD embryoid bodies treated with BMP2 for the first two days of differentiation. Data in all panels are represented as mean +/− SD and n = 3.

Treatment with BMP2 or Sox-17 during differentiation fully rescued the HDAC1-KD phenotype by repressing expression of pluripotency-associated genes after differentiation had been induced (Fig. 2B) and inducing expression of cardiomyocytes-associated genes (Fig. 3B–C). However, there are major steps to becoming a fully functional cardiomyocyte [15], [16]. First a cell needs to express cardiomyocyte specific markers committing to the lineage. In order to fully mature into a functional cardiomyocyte the cell needs to express mature cardiomyocyte markers that allow it to beat in synchrony and respond to external stimuli. We checked calcium transients in HDAC1-KD cells treated with BMP2 and assessed their ability to beat in synchrony and respond to external stimuli. HDAC1-KD-ES cells never beat, even when electrically stimulated (Fig. 4A), and do not have a calcium transient. However beating EBs derived from these cells when they were treated with BMP2, showed synchrony in beating as well as response to external stimuli similar to the wild type cells (Fig. 4D–E). Expression levels of a gap junction protein, Connexin 43 (CX-43) in these cells is also restored to levels similar to wild type and properly organized in the cell periphery within beating regions (Fig. 4F–G). Because mES-HDAC1-KD cells that had been treated with BMP2 show normal levels and peripheral expression pattern of CX-43 similar to the wild type, our data suggests that the aberrant pattern of expression of CX-43 in HDAC1-KD cells occurs because of the cells inability to turn on the Sox-17-BMP2 pathway in the absence of HDAC1, resulting in only partial differentiation. The ability of Sox-17 and BMP2 alone to fully rescue the HDAC1 KD phenotype in ES cells indicates that HDAC1 is crucial at the switching point in differentiation but once expression of Sox-17 is achieved in ES cells, they can differentiate to the cardiovascular phenotype, normally.

**Figure 5:**
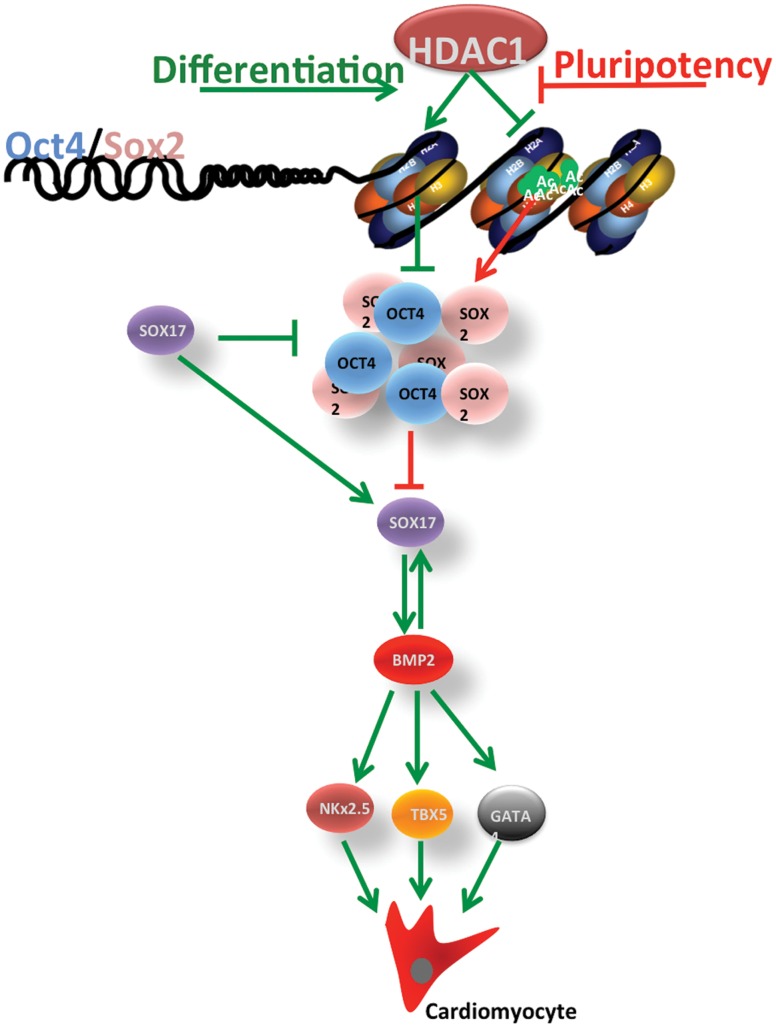
Model: HDAC1 is crucial during early differentiation . Loss of HDAC1 in pluripotent cells inhibits their ability to differentiate by suppressing the histone deacetylation of promoters of pluripotency associated genes, therefore resulting in their sustained expression and as a consequence repressed lineage specific differentiation. The prolonged expression of these pluripotency-associated genes results in continuous repression of Sox-17 which in turn in needed to induce expression of BMP2 in differentiating cells. As BMP2 is not expressed, expression of key cardiovascular transcription factors is not achieved, resulting in reduced or absent cardiovascular differentiation.

In summary, our data indicates that loss of HDAC1 in mES cells inhibits their ability to differentiate into the cardiomyocyte lineage. HDAC1 is important in these cells to suppress pluripotency-associate gene expression through deacetylation once differentiation has been induced. The prolonged expression of these pluripotency-associated genes results in continuous repression of Sox-17 which in turn is needed to induce expression of BMP2 in differentiating cells. As BMP2 is not expressed, expression of key cardiovascular transcription factors is not achieved, resulting in reduced or absent cardiovascular differentiation (model depicted in Figure 5). Other reports have indicated a key role for HDACs in ES cell differentiation through global inhibition of HDACs using Trichostatin A (TSA) [9], [10]. Although these reports have greatly extended the body of knowledge around histone deacetylases and differentiation, they were not designed to recognize and determine crucial differences between different members of the HDAC family. This may explain contradicting reports on the role of HDACs in inhibiting differentiation [10], [17] or in promoting differentiation [18] as both could be possible through different HDACs or different complexes they associate with [4], [19], [20]. Our data using HDAC1-KD pluripotent cells suggests that HDAC1 is specifically important in early differentiation as it is required to deacetylate puripotency- associated genes when differentiation is induced.

Additionally, expression of OCT4 and SOX2 has been shown to affect early differentiation genes such as SOX-17 expression [11], [12], [21] and expression of SOX-17 has been shown to be critical in cardiovascular differentiation [14] We propose a pathway through which chromatin modifications and expression of SOX-17 and cardiovascular genes are linked and are dependent on each other. Application of these pathways to the ES and iPS system explains and further supports the importance of SOX-17 and BMP2 in cardiovascular differentiation and provides an explanation of how HDAC1 controls cell fate into this particular lineage.

Lack of HDAC1 during differentiation results in reduced cardiovascular differentiation. Previous reports have indicated that HDAC1 knock out mice are embryonic lethal [7], [8]. However, cardiomyocyte restricted knock out of this gene (under the alpha-MHC promoter) has no effect on the phenotype [8]. We believe this is because HDAC1 is important very early in differentiation into cardiovascular lineages and alpha-MHC, being a protein expressed later in the development process, bypasses the state when HDAC1 is essential. In fact supplementing with proteins important early on in differentiation such as SOX-17 and BMP2 fully rescues the HDAC1 phenotype. Similarly, a cardiac restricted knock out under the alpha-MHC promoter missed the window of time in which we show HDAC1 to be important in affecting cardiovascular differentiation.

While mES-HDAC1-KD cells do not show any spontaneous beating during differentiation, synchronious beating as well as response to external stimuli are fully restored in these cells when treated with BMP2 or through exogenous overexpression of Sox-17. The arrhythmia observed in HDAC1/HDAC2 double cardiac specific-KO mice [8] could indicate an additive rather than redundant role for these enzymes in the heart.

Epigenetic molecular mechanisms important in maintaining pluripotency are crucial to our understanding of what makes pluripotent cells pluripotent and what governs their differentiation [22]–[25]. This body of knowledge, in the future, could lead to the development of a better translational strategy for the use of these powerful cells in regenerative medicine, including post-injury cardiovascular repair and regeneration.

## Materials and Methods

### Cell Types and Cell Culture

C57BL/6J murine ES cells were purchased from ATCC (Cat.# SCRC-1002) and were cultured in 15% FBS, 50 uM Beta-Mercaptoethanol, 1 mM nonessential amino acids and 100 U/ml Pen/Strep supplemented Dulbeco’s modified eagle medium (DMEM) in the presence of Leukemia Inhibitory factor (LIF; 10 ng/mL) as reported earlier [26].

### Formation of Embryoid Bodies (EB)

Differentiation of iPS and ES cells through embryoid body formation was performed using standard hanging drop method. Briefly, a single-cell suspension of each cell line at a concentration of 2.5×10^5^ cell/mL in 20 mL of differentiating media (Iscove’s Modified Dulbecco’s Medium (IDDM) supplemented with 15% FBS, 100 U/ml Pen/Strep, 200 ug/ml transferrin, 0.5 mML-ascorbic acid and 4.5×10^–4^ M monothioglycerol) was deposited in 20 ul hanging drops in 100×100 mm square petri dishes. After 2 days of being cultured in suspension, the cells were plated onto 0.1% gelatin coated dishes for continued differentiation.

### Sox-17 Over Expression

Sox-17 was over expressed in mES-HDAC1-KD cells using plasmids containing a Sox-17 expression cassette. Stable over-expression cell colonies were creating using G418 resistance selection.

### Real-Time Arrays and mRNA Expression

Expression analysis of epigenetic modifying enzymes and factors was performed using SABiosciences’s RT2 Profiler™ PCR Array System according to manufacturer’s instructions.

### HDAC1 Knock Down

The lentiviral-Hdac1 shRNA vectors were purchased from Sigma-Aldrich® and transduction was performed according to manufacturer’s instructions. Puromycin was used for selection.

### Immunofluorescence Staining

Protein expression analysis through immunofluorescence staining was performed as described earlier [26].

### Ca++ Studies

Calcium studies were performed mostly as previously described [27].

### Statistical Analysis

Two-way ANOVA followed by a Bonferroni post-hoc test was used to analyze the data. P values of <0.05 were used to determine significance.

## Supporting Information

Video S1 **Loss of HDAC1inhibits spontaneous cardiomyocyte differentiation in mES cells:** Embryoid Bodies derived from mES-HDAC1-KD show completely absent spontaneous beating (mES-HDAC1-KD) during differentiation. EBs derived from: mES wt cells (Video S1) and mES-HDAC1-KD cells (Video S2).
(MOV)

Video S2 **Loss of HDAC1inhibits spontaneous cardiomyocyte differentiation in mES cells:** Embryoid Bodies derived from mES-HDAC1-KD show completely absent spontaneous beating (mES-HDAC1-KD) during differentiation. EBs derived from: mES wt cells (Video S1) and mES-HDAC1-KD cells (Video S2).
(MOV)

Video S3 **Over-expression of Sox-17 rescues mES-HDAC1-KD phenotype:** Embryoid Bodies derived from mES-HDAC1-KD with ectopic expression of Sox-17 (Video S3) or treated with BMP2 (Video S4), during differentiation.
(MOV)

Video S4 **Over-expression of Sox-17 rescues mES-HDAC1-KD phenotype:** Embryoid Bodies derived from mES-HDAC1-KD with ectopic expression of Sox-17 (Video S3) or treated with BMP2 (Video S4), during differentiation.
(MOV)

## References

[1] Thomson JA, Itskovitz-Eldor J, Shapiro SS, Waknitz MA, Swiergiel JJ, et al (1998) Embryonic stem cell lines derived from human blastocysts. Science 282: 1145–1147. 9804556 10.1126/science.282.5391.1145

[2] Bernstein BE, Mikkelsen TS, Xie X, Kamal M, Huebert DJ, et al (2006) A bivalent chromatin structure marks key developmental genes in embryonic stem cells. Cell 125: 315–326. 16630819 10.1016/j.cell.2006.02.041

[3] Mikkelsen TS, Ku M, Jaffe DB, Issac B, Lieberman E, et al (2007) Genome-wide maps of chromatin state in pluripotent and lineage-committed cells. Nature 448: 553–560. 17603471 10.1038/nature06008 PMC2921165

[4] Liang J, Wan M, Zhang Y, Gu P, Xin H, et al (2008) Nanog and Oct4 associate with unique transcriptional repression complexes in embryonic stem cells. Nat Cell Biol 10: 731–739. 18454139 10.1038/ncb1736

[5] Ben-David U, Kopper O, Benvenisty N (2012) Expanding the boundaries of embryonic stem cells. Cell Stem Cell 10: 666–677. 22704506 10.1016/j.stem.2012.05.003

[6] Takahashi K, Yamanaka S (2006) Induction of pluripotent stem cells from mouse embryonic and adult fibroblast cultures by defined factors. Cell 126: 663–676. 16904174 10.1016/j.cell.2006.07.024

[7] Ma P, Schultz RM (2008) Histone deacetylase 1 (HDAC1) regulates histone acetylation, development, and gene expression in preimplantation mouse embryos. Dev Biol 319: 110–120. 18501342 10.1016/j.ydbio.2008.04.011 PMC2475681

[8] Montgomery RL, Davis CA, Potthoff MJ, Haberland M, Fielitz J, et al (2007) Histone deacetylases 1 and 2 redundantly regulate cardiac morphogenesis, growth, and contractility. Genes Dev 21: 1790–1802. 17639084 10.1101/gad.1563807 PMC1920173

[9] Karantzali E, Schulz H, Hummel O, Hubner N, Hatzopoulos A, et al (2008) Histone deacetylase inhibition accelerates the early events of stem cell differentiation: transcriptomic and epigenetic analysis. Genome Biol 9: R65. 18394158 10.1186/gb-2008-9-4-r65 PMC2643936

[10] Lee JH, Hart SR, Skalnik DG (2004) Histone deacetylase activity is required for embryonic stem cell differentiation. Genesis 38: 32–38. 14755802 10.1002/gene.10250

[11] Chen X, Xu H, Yuan P, Fang F, Huss M, et al (2008) Integration of external signaling pathways with the core transcriptional network in embryonic stem cells. Cell 133: 1106–1117. 18555785 10.1016/j.cell.2008.04.043

[12] Stefanovic S, Abboud N, Desilets S, Nury D, Cowan C, et al (2009) Interplay of Oct4 with Sox2 and Sox17: a molecular switch from stem cell pluripotency to specifying a cardiac fate. J Cell Biol 186: 665–673. 19736317 10.1083/jcb.200901040 PMC2742180

[13] Wang J, Rao S, Chu J, Shen X, Levasseur DN, et al (2006) A protein interaction network for pluripotency of embryonic stem cells. Nature 444: 364–368. 17093407 10.1038/nature05284

[14] Liu Y, Asakura M, Inoue H, Nakamura T, Sano M, et al (2007) Sox17 is essential for the specification of cardiac mesoderm in embryonic stem cells. Proc Natl Acad Sci U S A 104: 3859–3864. 17360443 10.1073/pnas.0609100104 PMC1820674

[15] Joggerst SJ, Hatzopoulos AK (2009) Stem cell therapy for cardiac repair: benefits and barriers. Expert Rev Mol Med 11: e20. 19586557 10.1017/S1462399409001124

[16] Hinkel R, Trenkwalder T, Kupatt C (2011) Gene therapy for ischemic heart disease. Expert Opin Biol Ther 11: 723–737. 21434842 10.1517/14712598.2011.570749

[17] Lagger S, Meunier D, Mikula M, Brunmeir R, Schlederer M, et al (2010) Crucial function of histone deacetylase 1 for differentiation of teratomas in mice and humans. EMBO J 29: 3992–4007. 20967026 10.1038/emboj.2010.264 PMC3020644

[18] Dovey OM, Foster CT, Cowley SM (2010) Histone deacetylase 1 (HDAC1), but not HDAC2, controls embryonic stem cell differentiation. Proc Natl Acad Sci U S A 107: 8242–8247. 20404188 10.1073/pnas.1000478107 PMC2889513

[19] Lee TI, Jenner RG, Boyer LA, Guenther MG, Levine SS, et al (2006) Control of developmental regulators by Polycomb in human embryonic stem cells. Cell 125: 301–313. 16630818 10.1016/j.cell.2006.02.043 PMC3773330

[20] Ren X (2006) Comments on control of developmental regulators by polycomb in human embryonic stem cells. Med Hypotheses 67: 1469–1470. 16919398 10.1016/j.mehy.2006.06.021

[21] Campbell PA, Perez-Iratxeta C, Andrade-Navarro MA, Rudnicki MA (2007) Oct4 targets regulatory nodes to modulate stem cell function. PLoS One 2: e553. 17579724 10.1371/journal.pone.0000553 PMC1891092

[22] Marmorstein R (2001) Protein modules that manipulate histone tails for chromatin regulation. Nat Rev Mol Cell Biol 2: 422–432. 11389466 10.1038/35073047

[23] Kim K, Doi A, Wen B, Ng K, Zhao R, et al (2010) Epigenetic memory in induced pluripotent stem cells. Nature 467: 285–290. 20644535 10.1038/nature09342 PMC3150836

[24] Narazaki G, Uosaki H, Teranishi M, Okita K, Kim B, et al (2008) Directed and systematic differentiation of cardiovascular cells from mouse induced pluripotent stem cells. Circulation 118: 498–506. 18625891 10.1161/CIRCULATIONAHA.108.769562

[25] Rolletschek A, Wobus AM (2009) Induced human pluripotent stem cells: promises and open questions. Biol Chem 390: 845–849. 19558327 10.1515/BC.2009.103

[26] Rajasingh J, Lambers E, Hamada H, Bord E, Thorne T, et al (2008) Cell-free embryonic stem cell extract-mediated derivation of multipotent stem cells from NIH3T3 fibroblasts for functional and anatomical ischemic tissue repair. Circ Res 102: e107–117. 18483406 10.1161/CIRCRESAHA.108.176115 PMC2435186

[27] Wasserstrom JA, Sharma R, Kapur S, Kelly JE, Kadish AH, et al (2009) Multiple defects in intracellular calcium cycling in whole failing rat heart. Circ Heart Fail 2: 223–232. 19808344 10.1161/CIRCHEARTFAILURE.108.811539

